# An Essay on the Antiquity of Hindoo Medicine, Including an Introductory Lecture to the Course of Materia Medica and Therapeutics Delivered at King's College

**Published:** 1838-10

**Authors:** 


### Art. XVI.

*An Essay on the Antiquity of Hindoo Medicine, including an Introductory Lecture to the Course of Materia Medica and Therapeutics delivered at King's College.* By J. F. Royle, m.d. f.r. & l.s. &c., Professor of Materia Medica King's College.—*London,* 1837. 8vo. p. 196.

This work of Dr. Royle's exhibits considerable research in ground hitherto almost untrodden, and opens entirely new views on the origin and progress of medicine during the early ages. In this respect it is a very creditable addition to the few and unsatisfactory notices we possess on the early history of medicine. It is also particularly acceptable for the accuracy of its details on the geographical distribution of some of our most valuable drugs. The industry with which the investigation of this subject has been pursued may be appreciated from the following statement.

"It would be tedious to relate the various measures I adopted for acquiring a knowledge of the substances at present in use in the several parts of that extensive empire (India); as well as for pointing out the sources whence a still greater variety might, if desirable, be obtained. It is enough to say, that I made collections of every thing that was procurable in their bazaars, tracing them as much as possible to the plants, animals, and countries, whence they were derived. I had the native works on Materia Medica collated by competent Hakeems and Moonshees, and the several articles arranged under the three heads of the animal, vegetable, and mineral kingdoms." (p. 25.)

From the facts which Dr. Royle has collected together, it is very evident that both the Arabians and Persians obtained from Northern India many of the articles of their materia medica; and that they also availed themselves of the written experience of the Hindoos. This we are not altogether surprised at, but we were not prepared to find that many of the plumes of originality are plucked from their more illustrious predecessors, the Greeks.

No certain period can be assigned to the Hindoo authors on materia medica; "for these, like the writers on every other subject among this extraordinary people, are without any other than their fabulous chronology, by which we may even approximatively ascertain the age in which they lived." It is, however, made very evident that medical science and the knowledge both of mineral and vegetable medicines, was in a state of considerable advancement among this people even anterior to the time of the so-called father of physic himself. That this ancient high state of Hindoo medical learning, attended as it was by the extensive disseminating of the indigenous articles of its materia medica, has not found record in the former annals of medicine, can only be explained by the total ignorance, which has prevailed in Europe, even to our own day, of the refined language of India.

"For when the name of even the most celebrated Hindoo writer presented itself before a modern author writing expressly on the subject, it is passed over without comment or examination—'Scharak Indus, a Rhazeo citatus plane ignotus." (Sprengel. Hist. Rei. Herb. 1. p. 234.) That a Hindoo system of medicine does exist, we know from their numerous books on all branches of that science; that some of these were written prior to the Arabs, we have shown by their being quoted in the works of the latter. How much earlier than the eighth century the principal of them were composed, we may only hope to ascertain by the progress now making in settling Indian Chronology. But in absence of this it is practicable, as I have stated, to get a conviction of the cultivation of medicine among the Hindoos at still earlier periods, from occasional notices by writers of the West; and we cannot but allow them an early knowledge of the properties of many of the valuable drugs which their country afforded them, when we see the necessarily subsequent employment of the same by the Greeks and Romans." (p. 75.)

Dr. Royle has very ingeniously brought together much collateral evidence to show the great antiquity of Hindoo medicine. He shows that the work of Dioscorides, who lived A.D. 63, is well calculated to prove how much the ancients were indebted to India and the East for their medicines. Dioscorides has however fallen into the error of stating many drugs to be the natural productions of Syria and Media, which are indigenous only to India; but this is easily explained by our knowing that the products of the East reached the West both by the Red Sea, Arabia, and Egypt; as also by the Euphrates, through the desert-surrounded Palmyra to Syria: he has therefore considered the place of export as the country actually producing the drug. Theophrastus (who lived B. C. 288,) mentions many of the spices and aromatics of India, as does also Hippocrates. But a yet more remote antiquity may be assigned to medical knowledge amongst these people, if we consider the period when translations were made from Hindoo works into other languages, though this indicates rather the age when neighbouring nations were able to appreciate the value of such works than the time when they were written. "Thus they were translated into Tamul by the Maha Rishi Aghastier, who appears to have introduced the religion as well as the science of the Hindoos into the Peninsula before the Christian era." (p. 66.)

The extent of their knowledge may be appreciated from the nature of their therapeutics:

"But in the works of Charak and Susruta, to which, as has been proved, the earliest of the Arabs had access, we find numerous metallic substances directed to be given internally; as oxide of iron, with ginger and cinnamon as a tonic; the rust in cachexy, and the sulphate in dropsy. Arsenic they prescribed not only in leprosy, and probably other cutaneous affections, but the oxide (arsenious acid) has long been with them a favorite and most efficacious remedy, in conjunction with pepper and aromatics, for the cure of intermittent fevers. Mercury appears to have been externally employed in the time of Pliny, as in his work occurs the remarkable passage (lib. xxxiii. cap. 8): 'Omnia quæ de minio in medicinæ usu traduntur, temeraria arbitror: præterquam fortassis illito capite ventreve sanguinem sistendum, dum ne quid penetret in viscera, ac vulnus attingat: aliter utendum non equidem censeam.' But the Hindoos have from very early times been in the habit of prescribing the sulphuret in the form of fumigation; and the preparations which we consider equivalent to calomel and to corrosive sublimate, in the form of pills combined with sugar, pepper, and aromatics; in a great variety of affections and to the extent of exciting salivation." (p. 45.)

It appears that the ancient Hindoos everywhere except in large towns practised all branches of the profession, as is the custom amongst the moderns. Those who practised surgery appear to have been acquainted with some of the more important operations, as lithotomy and extraction of the fœtus *ex utero*, and in their works are descriptions of no less than 127 surgical instruments.

The following observations upon theoretical and practical physicians, promulgated in one of their earliest works may be read with advantage even in the present day.

"'Having completed the indispensable course of study, practice is then to be as indispensably acquired; for he, who is versed in both, deserves to be honoured as the chief of physicians. As it is said, he who is acquainted with the science of medicine only from studying the books which treat of it, and is not well grounded in the practice also, is bewildered when called upon to attend the sick, like a coward in the day of battle. He who engages in practice, presumptuously disregarding written science, is held in no estimation by the virtuous, and merits death from the King. Both these descriptions of persons are unskilful, incompetent in their profession, possessed of only one branch of the necessary qualifications, like birds with but one wing. The medicaments, which contain within them the properties of ambrosia, are as sharp weapons, the deadly thunderbolt, or fatal poisons when administered by the ignorant; let no such man be trusted. He, who, imperfectly master of his profession, treats maladies which require either medicines or the knife, murders his patients—the vile practitioner! through his own cupidity, and the fault of the ruling authority. But he, who is conversant with both theory and practice, is competent to attain the object of his professional career, borne onwards like a war-chariot on two wheels through the ranks of the enemy.'" (p. 49.)

Amid much of the good sense with which the administration of medicines is recommended, a compliance with various ceremonies is directed which may in the present day excite a smile; after these have however been complied with, it is quaintly recommended for the patient's good that before he takes the medicine "the god of physic is to be worshipped, in the person of his deputy, the physician, who must be paid well for his services."

We recommend Dr. Royle's learned yet unpretending volume to the notice of all interested in the study of the history of medicine and the materia medica.

